# Crystal structure of (3,5-di­chloro-2-hy­droxy­phen­yl){1-[(naphthalen-1-yl)carbon­yl]-1*H*-pyrazol-4-yl}methanone

**DOI:** 10.1107/S1600536814024684

**Published:** 2014-11-19

**Authors:** Yoshinobu Ishikawa, Yuya Motohashi

**Affiliations:** a School of Pharmaceutical Sciences, University of Shizuoka, 52-1 Yada, Suruga-ku, Shizuoka 422-8526, Japan

**Keywords:** crystal structure, diaroyl pyrazole, cyclization, stacking inter­action, C—H⋯O hydrogen bonding

## Abstract

The title compound, C_21_H_12_Cl_2_N_2_O_3_, is a 1,4-diaroyl pyrazole derivative and has three aromatic rings. The dihedral angles between the naphthalene ring system and the pyrazole ring, the pyrazole and phenyl rings and the naphthalene ring system and the phenyl ring are 49.44 (13), 49.87 (16) and 0.58 (11)°, respectively. The phenolic proton forms an intra­molecular O—H⋯O hydrogen bond with an adjacent carbonyl O atom. In the crystal, the mol­ecules are linked through stacking inter­actions between the pyrazole rings [centroid–centroid distances = 3.546 (3)] and between the naphthalene ring system and the phenyl ring [centroid–centroid distances = 3.609 (4) Å] along the *a*-axis direction. The mol­ecules are further connected through C—H⋯O hydrogen bonds, forming inversion dimers.

## Abstract 2

The title compound is a 1,4-diaroyl pyrazole derivative and has three aromatic rings. In the crystal, mol­ecules are linked through stacking inter­actions between the pyrazole rings and between the naphthalene and phenyl rings, and through inter­molecular C—H⋯O hydrogen bonds to form inversion dimers.

## Chemical context   

3-Formyl­chromones are used as diverse building blocks (Ali *et al.*, 2013 ▶), and their Schiff base derivatives have attracted much attention in medicinal chemistry (Nawrot-Modranka *et al.*, 2006 ▶; Khan *et al.*, 2009 ▶; Wang *et al.*, 2008 ▶; Tu *et al.*, 2013 ▶; Gaspar *et al.*, 2014 ▶). We have recently reported the crystal structures of such Schiff base compounds (Ishikawa & Watanabe, 2014*a* ▶,*b* ▶,*c* ▶,*d* ▶), which were prepared from condensation reactions of 3-formyl­chromones with aryl­hydrazides. Inter­estingly, crystallographic analysis revealed that the structure of the orange crystals obtained from crystallization of the white solid prepared from the condensation reaction of 6,8-di­bromo-3-formyl­chromone (Ishikawa, 2014 ▶) with 1-naph­tho­hydrazide is a 1,4-diaroyl pyrazole (Ishikawa & Motohashi, 2014 ▶).
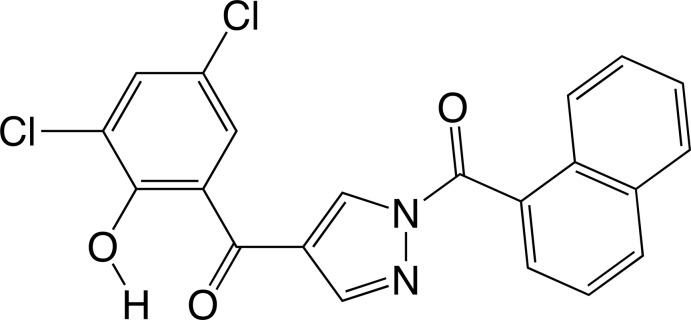


## Structural commentary   

**Figure 1:**
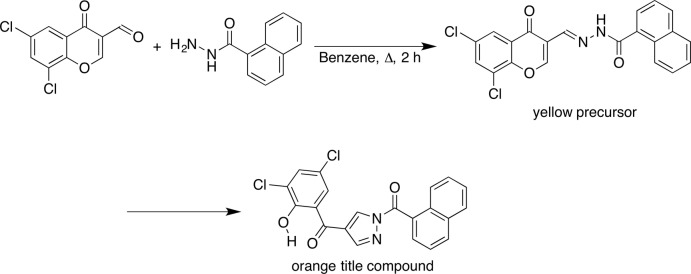
Reaction scheme for the title compound.

**Figure 2:**
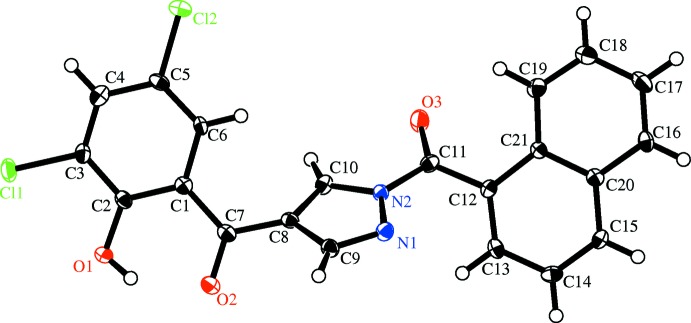
The mol­ecular structure of the title compound, with displacement ellipsoids drawn at the 50% probability level. Hydrogen atoms are shown as small spheres of arbitrary radius.

The reaction of 6,8-di­chloro-3-formyl­chromone (Ishikawa & Motohashi, 2013 ▶) with 1-naphtho­ylhydrazide in benzene gave yellow solids, and orange crystals were obtained from an ethyl acetate/acetone solution of the yellow solids (Fig. 1 ▶). The crystallographic analysis revealed that the structure of the orange crystals is a 1,4-diaroyl pyrazole, as shown in Fig. 2 ▶, which should be thermodynamically more stable than that of the yellow solids. The dihedral angles between the naphthalene ring system and the pyrazole ring, the pyrazole and phenyl rings and the naphthalene ring system and the phenyl ring are 49.44 (13), 49.87 (16) and 0.58 (11)°, respectively. The phenolic proton forms an intra­molecular O–H⋯O hydrogen bond with the adjacent carbonyl O2 atom. The conformation of the title compound is almost identical to that of our previously reported 1,4-diaroyl pyrazole derivative (Ishikawa & Motohashi, 2014 ▶).

The driving force of the intra­molecular cyclization (Fig. 1 ▶) should be a resonance energy gain, resulting from the extension of the conjugated system across the entire mol­ecule. The intra­molecular cyclization is not observed for the chromone derivatives without electron-withdrawing substituents (Ishikawa & Watanabe, 2014*a* ▶,*b* ▶,*c* ▶,*d* ▶), and thus the activation energy for the chromone derivative with the electron-withdrawing substituents should be lower than that for ones without electron-withdrawing substituents.

## Supra­molecular features   

**Figure 3:**
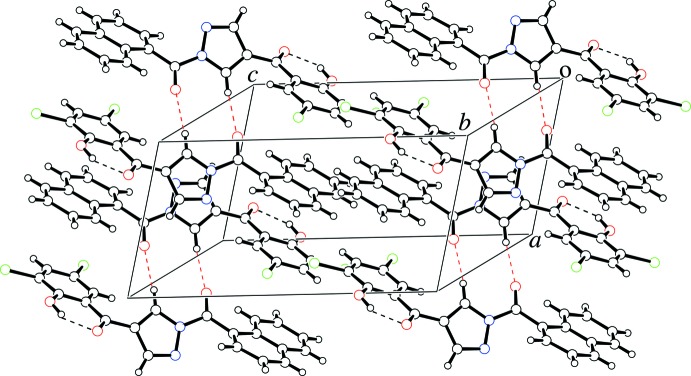
A crystal packing view of the title compound. Intra­molecular O—H⋯O and inter­molecular C—H⋯O hydrogen bonds are represented by black and red dashed lines, respectively.

The mol­ecules are linked along the *a*-axis through stacking inter­actions between inversion-related pyrazole rings, and between the naphthalene ring system and the phenyl ring of an inversion-related molecule [centroid–centroid distances = 3.546 (3) and 3.609 (4) Å, respectively; symmetry code: –*x* + 1, –*y* + 1, –*z*]. The mol­ecules are further connected through inter­molecular C—H⋯O hydrogen bonds (Table 1 ▶), forming inversion dimers, as shown in Fig. 3 ▶. Type I halogen⋯halogen contacts between the chlorine atoms, which is seen in the crystal structure of the starting material, 6,8-di­chloro-3-formyl­chromone (Ishikawa & Motohashi, 2013 ▶), are not observed.

## Database survey   

In the WebCSD (Version 1.1.1, last update November 2014; Groom & Allen, 2014 ▶) no structures of compounds containing a 1,4-diaroyl pyrazole entity are listed except our previously reported one (Ishikawa & Motohashi, 2014 ▶).

## Synthesis and crystallization   

Preparation of the yellow precursor, (*E*)-*N*′-[(6,8-di­chloro-4-oxo-4*H*-chromen-3-yl)methyl­ene]-1-naphtho­hydrazide, is as follows: 1-naphtho­hydrazide (2.7 mmol) and 6,8-di­chloro-3-formyl­chromone (2.7 mmol) were dissolved in 50 ml of benzene, and the mixture was refluxed with a Dean–Stark apparatus for 2 h with stirring. After cooling, the yellow precipitates were collected, washed with *n*-hexane and dried *in vacuo* (yield 18%). ^1^H NMR (400 MHz, DMSO-*d*_6_): *δ* = 7.60–7.64 (*m*, 4H), 7.78 (*d*, 1H, *J* = 6.9 Hz), 8.03 (*d*, 1H, *J* = 2.5 Hz), 8.11 (*d*, 1H, *J* = 8.3 Hz), 8.23 (*m*, 1H), 8.26 (*d*, 1H, *J* = 2.5 Hz), 8.48 (*s*, 1H), 8.98 (*s*, 1H), 12.17 (*s*, 1H). DART–MS calculated for [C_21_H_12_Cl_2_N_2_O_3_ + H^+^]: 411.030, found 410.905. Orange crystals of the title compound suitable for X-ray diffraction were obtained by slow evaporation of an ethyl acetate/acetone solution of the yellow precursor at room temperature.

## Refinement   

Crystal data, data collection and structure refinement details are summarized in Table 2 ▶. The C-bound hydrogen atoms were placed in geometrical positions and refined using a riding model [C—H 0.95 Å with *U*_iso_(H) = 1.2*U*_eq_(C)]. The phenolic proton was located in a difference Fourier map, and refined using a riding model [O—H 0.84 Å with *U*_iso_(H) = 1.5*U*_eq_(O)].

## Supplementary Material

Crystal structure: contains datablock(s) General, I. DOI: 10.1107/S1600536814024684/hb7275sup1.cif

Structure factors: contains datablock(s) I. DOI: 10.1107/S1600536814024684/hb7275Isup2.hkl

Supporting information file. DOI: 10.1107/S1600536814024684/hb7275Isup3.cml

CCDC reference: 1033534

Additional supporting information:  crystallographic information; 3D view; checkCIF report

## Figures and Tables

**Table 1:** Hydrogen-bond geometry (, )

*D*H*A*	*D*H	H*A*	*D* *A*	*D*H*A*
O1H3O2	0.84	1.84	2.570(4)	144
C10H5O3^i^	0.95	2.29	3.219(6)	166

Symmetry code: (i) 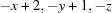.

**Table 2:** Experimental details

Crystal data	
Chemical formula	C_21_H_12_Cl_2_N_2_O_3_
*M*_r_	411.24
Crystal system, space group	Triclinic, *P* 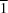
Temperature (K)	100
*a*, *b*, *c* ()	7.342(7), 8.807(4), 14.861(5)
, , ()	75.49(3), 76.88(5), 70.51(5)
*V* (^3^)	866.1(9)
*Z*	2
Radiation type	Mo *K*
(mm^1^)	0.40
Crystal size (mm)	0.40 0.12 0.05

Data collection	
Diffractometer	Rigaku AFC-7R diffractometer
No. of measured, independent and observed [*F* ^2^ > 2(*F* ^2^)] reflections	4892, 3992, 2316
*R*_int_	0.051
(sin /)_max_ (^1^)	0.650

Refinement	
*R*[*F* ^2^ > 2(*F* ^2^)], *wR*(*F* ^2^), *S*	0.059, 0.163, 1.01
No. of reflections	3992
No. of parameters	254
H-atom treatment	H-atom parameters constrained
_max_, _min_ (e ^3^)	0.65, 0.57

Computer programs: *WinAFC* (Rigaku, 1999 ▶), *SIR2008* (Burla *et al.*, 2007 ▶), *SHELXL97* (Sheldrick, 2008 ▶), *CrystalStructure* (Rigaku, 2010 ▶).

## supplementary crystallographic information

### Crystal data

**Table:**

C_21_H_12_Cl_2_N_2_O_3_	*Z* = 2
*M**_r_* = 411.24	*F*(000) = 420.00
Triclinic, *P*1	*D*_x_ = 1.577 Mg m^−^^3^
Hall symbol: -P 1	Mo *K*α radiation, λ = 0.71069 Å
*a* = 7.342 (7) Å	Cell parameters from 15 reflections
*b* = 8.807 (4) Å	θ = 15.0–17.2°
*c* = 14.861 (5) Å	µ = 0.40 mm^−^^1^
α = 75.49 (3)°	*T* = 100 K
β = 76.88 (5)°	Plate, colorless
γ = 70.51 (5)°	0.40 × 0.12 × 0.05 mm
*V* = 866.1 (9) Å^3^	

### Data collection

**Table:**

Rigaku AFC-7R diffractometer	θ_max_ = 27.5°
ω–2θ scans	*h* = −9→5
4892 measured reflections	*k* = −11→10
3992 independent reflections	*l* = −19→18
2316 reflections with *F*^2^ > 2σ(*F*^2^)	3 standard reflections every 150 reflections
*R*_int_ = 0.051	intensity decay: 0.1%

### Refinement

**Table:**

Refinement on *F*^2^	Secondary atom site location: difference Fourier map
*R*[*F*^2^ > 2σ(*F*^2^)] = 0.059	Hydrogen site location: inferred from neighbouring sites
*wR*(*F*^2^) = 0.163	H-atom parameters constrained
*S* = 1.01	*w* = 1/[σ^2^(*F*_o_^2^) + (0.0752*P*)^2^] where *P* = (*F*_o_^2^ + 2*F*_c_^2^)/3
3992 reflections	(Δ/σ)_max_ < 0.001
254 parameters	Δρ_max_ = 0.65 e Å^−^^3^
0 restraints	Δρ_min_ = −0.57 e Å^−^^3^
Primary atom site location: structure-invariant direct methods	

### Special details

**Table:**

Refinement. Refinement was performed using all reflections. The weighted *R*-factor (*wR*) and goodness of fit (*S*) are based on *F*^2^. *R*-factor (gt) are based on *F*. The threshold expression of *F*^2^ > 2.0σ(*F*^2^) is used only for calculating *R*-factor (gt).

### Fractional atomic coordinates and isotropic or equivalent isotropic displacement parameters (Å^2^)

**Table:**

	*x*	*y*	*z*	*U*_iso_*/*U*_eq_	
Cl1	1.19340 (16)	0.01726 (12)	−0.42044 (7)	0.0251 (3)	
Cl2	0.99842 (15)	0.61336 (11)	−0.33214 (7)	0.0209 (3)	
O1	1.0166 (5)	−0.0798 (3)	−0.22817 (19)	0.0202 (7)	
O2	0.7994 (4)	−0.0183 (4)	−0.07181 (19)	0.0199 (6)	
O3	0.8243 (5)	0.5545 (4)	0.10106 (19)	0.0244 (7)	
N1	0.5085 (5)	0.3295 (4)	0.1019 (3)	0.0175 (7)	
N2	0.6723 (5)	0.3817 (4)	0.0846 (2)	0.0140 (7)	
C1	0.9146 (6)	0.1862 (5)	−0.1837 (3)	0.0145 (8)	
C2	1.0070 (6)	0.0793 (5)	−0.2486 (3)	0.0155 (8)	
C3	1.0896 (6)	0.1441 (5)	−0.3387 (3)	0.0162 (8)	
C4	1.0860 (6)	0.3057 (5)	−0.3643 (3)	0.0189 (9)	
C5	0.9990 (6)	0.4094 (5)	−0.2991 (3)	0.0161 (8)	
C6	0.9122 (6)	0.3522 (5)	−0.2108 (3)	0.0149 (8)	
C7	0.8156 (6)	0.1231 (5)	−0.0911 (3)	0.0158 (8)	
C8	0.7275 (6)	0.2275 (5)	−0.0197 (3)	0.0145 (8)	
C9	0.5447 (6)	0.2354 (5)	0.0396 (3)	0.0177 (8)	
C10	0.8039 (6)	0.3241 (5)	0.0109 (3)	0.0169 (8)	
C11	0.7010 (6)	0.4863 (5)	0.1363 (3)	0.0167 (8)	
C12	0.5799 (6)	0.4989 (5)	0.2304 (3)	0.0138 (8)	
C13	0.5421 (6)	0.3616 (5)	0.2882 (3)	0.0154 (8)	
C14	0.4469 (6)	0.3640 (5)	0.3814 (3)	0.0173 (8)	
C15	0.3854 (6)	0.5064 (5)	0.4145 (3)	0.0162 (8)	
C16	0.3530 (6)	0.8001 (5)	0.3932 (3)	0.0170 (8)	
C17	0.3845 (7)	0.9404 (5)	0.3393 (3)	0.0236 (10)	
C18	0.4877 (6)	0.9396 (5)	0.2466 (3)	0.0208 (9)	
C19	0.5538 (6)	0.8003 (5)	0.2102 (3)	0.0188 (9)	
C20	0.4198 (6)	0.6516 (5)	0.3576 (3)	0.0148 (8)	
C21	0.5229 (6)	0.6510 (5)	0.2634 (3)	0.0135 (8)	
H1	1.1422	0.3469	−0.4259	0.0227*	
H2	0.8500	0.4248	−0.1678	0.0179*	
H3	0.9394	−0.0983	−0.1784	0.0243*	
H4	0.4580	0.1792	0.0349	0.0212*	
H5	0.9252	0.3466	−0.0144	0.0203*	
H6	0.5812	0.2626	0.2649	0.0185*	
H7	0.4255	0.2666	0.4211	0.0207*	
H8	0.3180	0.5081	0.4771	0.0195*	
H9	0.2853	0.8005	0.4557	0.0203*	
H10	0.3375	1.0389	0.3637	0.0284*	
H11	0.5112	1.0379	0.2091	0.0250*	
H12	0.6224	0.8032	0.1477	0.0225*	

### Atomic displacement parameters (Å^2^)

**Table:**

	*U*^11^	*U*^22^	*U*^33^	*U*^12^	*U*^13^	*U*^23^
Cl1	0.0301 (6)	0.0222 (5)	0.0197 (5)	−0.0007 (5)	−0.0007 (5)	−0.0105 (4)
Cl2	0.0231 (6)	0.0147 (5)	0.0260 (6)	−0.0075 (4)	−0.0069 (4)	−0.0004 (4)
O1	0.0260 (17)	0.0121 (13)	0.0198 (15)	−0.0047 (12)	0.0005 (12)	−0.0030 (11)
O2	0.0243 (15)	0.0159 (13)	0.0207 (15)	−0.0067 (12)	−0.0036 (12)	−0.0044 (11)
O3	0.0242 (16)	0.0317 (16)	0.0237 (16)	−0.0160 (14)	0.0035 (13)	−0.0125 (13)
N1	0.0177 (17)	0.0212 (17)	0.0156 (16)	−0.0079 (14)	−0.0031 (13)	−0.0039 (13)
N2	0.0146 (16)	0.0147 (15)	0.0137 (16)	−0.0051 (13)	−0.0026 (13)	−0.0033 (13)
C1	0.0121 (18)	0.0147 (18)	0.0162 (19)	−0.0013 (15)	−0.0060 (15)	−0.0018 (15)
C2	0.016 (2)	0.0129 (17)	0.0175 (19)	−0.0015 (15)	−0.0056 (16)	−0.0042 (15)
C3	0.016 (2)	0.0175 (19)	0.0137 (19)	−0.0011 (16)	−0.0035 (15)	−0.0051 (15)
C4	0.0142 (19)	0.024 (2)	0.016 (2)	−0.0042 (16)	−0.0032 (16)	−0.0018 (16)
C5	0.0149 (19)	0.0113 (17)	0.023 (2)	−0.0023 (15)	−0.0079 (16)	−0.0023 (15)
C6	0.0164 (19)	0.0145 (18)	0.0146 (18)	−0.0024 (15)	−0.0028 (15)	−0.0066 (15)
C7	0.017 (2)	0.0129 (17)	0.018 (2)	−0.0024 (15)	−0.0078 (16)	−0.0023 (15)
C8	0.0158 (19)	0.0128 (17)	0.0157 (19)	−0.0043 (15)	−0.0052 (15)	−0.0013 (14)
C9	0.022 (2)	0.0170 (19)	0.0165 (19)	−0.0064 (16)	−0.0062 (16)	−0.0037 (15)
C10	0.0140 (19)	0.0203 (19)	0.0146 (19)	−0.0029 (16)	−0.0022 (15)	−0.0029 (15)
C11	0.014 (2)	0.0187 (19)	0.0161 (19)	−0.0055 (16)	−0.0016 (15)	−0.0010 (15)
C12	0.0123 (18)	0.0177 (18)	0.0119 (18)	−0.0040 (15)	−0.0044 (14)	−0.0021 (14)
C13	0.0137 (19)	0.0167 (18)	0.0176 (19)	−0.0016 (15)	−0.0064 (15)	−0.0067 (15)
C14	0.016 (2)	0.0159 (18)	0.019 (2)	−0.0054 (16)	−0.0072 (16)	0.0027 (15)
C15	0.016 (2)	0.0204 (19)	0.0116 (18)	−0.0051 (16)	−0.0019 (15)	−0.0025 (15)
C16	0.017 (2)	0.0195 (19)	0.0165 (19)	−0.0029 (16)	−0.0046 (16)	−0.0080 (16)
C17	0.033 (3)	0.0147 (19)	0.026 (3)	−0.0036 (18)	−0.0139 (19)	−0.0059 (17)
C18	0.026 (3)	0.0180 (19)	0.022 (2)	−0.0077 (17)	−0.0117 (18)	−0.0009 (16)
C19	0.023 (3)	0.021 (2)	0.0137 (19)	−0.0076 (17)	−0.0084 (16)	−0.0004 (16)
C20	0.0135 (19)	0.0136 (18)	0.0161 (19)	−0.0006 (15)	−0.0065 (15)	−0.0016 (14)
C21	0.0138 (19)	0.0136 (17)	0.0142 (18)	−0.0030 (15)	−0.0056 (15)	−0.0026 (14)

### Geometric parameters (Å, º)

**Table:**

Cl1—C3	1.731 (5)	C13—C14	1.405 (6)
Cl2—C5	1.738 (4)	C14—C15	1.360 (6)
O1—C2	1.338 (5)	C15—C20	1.411 (6)
O2—C7	1.245 (5)	C16—C17	1.354 (6)
O3—C11	1.203 (6)	C16—C20	1.425 (6)
N1—N2	1.376 (6)	C17—C18	1.415 (6)
N1—C9	1.319 (6)	C18—C19	1.360 (7)
N2—C10	1.364 (5)	C19—C21	1.415 (6)
N2—C11	1.428 (7)	C20—C21	1.433 (5)
C1—C2	1.417 (6)	O1—H3	0.840
C1—C6	1.411 (6)	C4—H1	0.950
C1—C7	1.471 (5)	C6—H2	0.950
C2—C3	1.402 (5)	C9—H4	0.950
C3—C4	1.371 (6)	C10—H5	0.950
C4—C5	1.398 (6)	C13—H6	0.950
C5—C6	1.373 (5)	C14—H7	0.950
C7—C8	1.473 (6)	C15—H8	0.950
C8—C9	1.419 (6)	C16—H9	0.950
C8—C10	1.368 (7)	C17—H10	0.950
C11—C12	1.484 (5)	C18—H11	0.950
C12—C13	1.368 (6)	C19—H12	0.950
C12—C21	1.439 (6)		
			
N2—N1—C9	104.0 (3)	C14—C15—C20	121.2 (4)
N1—N2—C10	112.0 (4)	C17—C16—C20	121.1 (4)
N1—N2—C11	124.3 (3)	C16—C17—C18	119.6 (4)
C10—N2—C11	123.7 (4)	C17—C18—C19	120.9 (4)
C2—C1—C6	119.3 (4)	C18—C19—C21	121.6 (4)
C2—C1—C7	119.1 (4)	C15—C20—C16	120.5 (4)
C6—C1—C7	121.5 (4)	C15—C20—C21	120.2 (4)
O1—C2—C1	123.0 (4)	C16—C20—C21	119.3 (4)
O1—C2—C3	118.6 (4)	C12—C21—C19	125.9 (4)
C1—C2—C3	118.3 (4)	C12—C21—C20	116.6 (4)
Cl1—C3—C2	118.9 (3)	C19—C21—C20	117.4 (4)
Cl1—C3—C4	119.3 (3)	C2—O1—H3	109.475
C2—C3—C4	121.8 (4)	C3—C4—H1	120.231
C3—C4—C5	119.5 (4)	C5—C4—H1	120.248
Cl2—C5—C4	119.0 (3)	C1—C6—H2	119.839
Cl2—C5—C6	120.4 (3)	C5—C6—H2	119.826
C4—C5—C6	120.6 (4)	N1—C9—H4	123.764
C1—C6—C5	120.3 (4)	C8—C9—H4	123.759
O2—C7—C1	121.0 (4)	N2—C10—H5	126.593
O2—C7—C8	118.2 (4)	C8—C10—H5	126.577
C1—C7—C8	120.8 (4)	C12—C13—H6	119.300
C7—C8—C9	125.3 (5)	C14—C13—H6	119.300
C7—C8—C10	129.8 (4)	C13—C14—H7	120.180
C9—C8—C10	104.7 (4)	C15—C14—H7	120.174
N1—C9—C8	112.5 (5)	C14—C15—H8	119.408
N2—C10—C8	106.8 (4)	C20—C15—H8	119.412
O3—C11—N2	117.3 (4)	C17—C16—H9	119.434
O3—C11—C12	125.1 (5)	C20—C16—H9	119.430
N2—C11—C12	117.5 (4)	C16—C17—H10	120.195
C11—C12—C13	119.5 (4)	C18—C17—H10	120.201
C11—C12—C21	119.3 (4)	C17—C18—H11	119.540
C13—C12—C21	120.8 (4)	C19—C18—H11	119.536
C12—C13—C14	121.4 (4)	C18—C19—H12	119.210
C13—C14—C15	119.6 (4)	C21—C19—H12	119.207
			
H3—O1—C2—C1	13.1	C9—C8—C10—H5	−179.2
H3—O1—C2—C3	−165.9	C10—C8—C9—N1	0.3 (4)
N2—N1—C9—C8	−1.2 (4)	C10—C8—C9—H4	−179.7
N2—N1—C9—H4	178.8	O3—C11—C12—C13	−139.1 (4)
C9—N1—N2—C10	1.8 (4)	O3—C11—C12—C21	33.9 (6)
C9—N1—N2—C11	−179.2 (3)	N2—C11—C12—C13	39.3 (5)
N1—N2—C10—C8	−1.7 (4)	N2—C11—C12—C21	−147.7 (3)
N1—N2—C10—H5	178.3	C11—C12—C13—C14	172.9 (4)
N1—N2—C11—O3	−162.4 (3)	C11—C12—C13—H6	−7.1
N1—N2—C11—C12	19.1 (5)	C11—C12—C21—C19	7.2 (6)
C10—N2—C11—O3	16.5 (5)	C11—C12—C21—C20	−175.0 (4)
C10—N2—C11—C12	−162.0 (3)	C13—C12—C21—C19	−180.0 (4)
C11—N2—C10—C8	179.3 (3)	C13—C12—C21—C20	−2.2 (6)
C11—N2—C10—H5	−0.7	C21—C12—C13—C14	0.0 (6)
C2—C1—C6—C5	−0.3 (6)	C21—C12—C13—H6	−180.0
C2—C1—C6—H2	179.7	C12—C13—C14—C15	1.9 (6)
C6—C1—C2—O1	179.6 (4)	C12—C13—C14—H7	−178.1
C6—C1—C2—C3	−1.3 (6)	H6—C13—C14—C15	−178.1
C2—C1—C7—O2	−5.4 (6)	H6—C13—C14—H7	1.9
C2—C1—C7—C8	176.5 (4)	C13—C14—C15—C20	−1.6 (7)
C7—C1—C2—O1	−3.1 (6)	C13—C14—C15—H8	178.4
C7—C1—C2—C3	175.9 (4)	H7—C14—C15—C20	178.4
C6—C1—C7—O2	171.7 (4)	H7—C14—C15—H8	−1.6
C6—C1—C7—C8	−6.3 (6)	C14—C15—C20—C16	179.5 (4)
C7—C1—C6—C5	−177.4 (4)	C14—C15—C20—C21	−0.6 (6)
C7—C1—C6—H2	2.6	H8—C15—C20—C16	−0.5
O1—C2—C3—Cl1	1.9 (6)	H8—C15—C20—C21	179.4
O1—C2—C3—C4	−179.7 (4)	C17—C16—C20—C15	−179.8 (4)
C1—C2—C3—Cl1	−177.2 (4)	C17—C16—C20—C21	0.3 (7)
C1—C2—C3—C4	1.2 (7)	C20—C16—C17—C18	−0.9 (7)
Cl1—C3—C4—C5	178.8 (3)	C20—C16—C17—H10	179.1
Cl1—C3—C4—H1	−1.2	H9—C16—C17—C18	179.1
C2—C3—C4—C5	0.4 (7)	H9—C16—C17—H10	−0.9
C2—C3—C4—H1	−179.6	H9—C16—C20—C15	0.2
C3—C4—C5—Cl2	179.2 (4)	H9—C16—C20—C21	−179.7
C3—C4—C5—C6	−2.0 (7)	C16—C17—C18—C19	0.8 (7)
H1—C4—C5—Cl2	−0.8	C16—C17—C18—H11	−179.2
H1—C4—C5—C6	178.0	H10—C17—C18—C19	−179.2
Cl2—C5—C6—C1	−179.3 (3)	H10—C17—C18—H11	0.8
Cl2—C5—C6—H2	0.7	C17—C18—C19—C21	−0.2 (7)
C4—C5—C6—C1	1.9 (7)	C17—C18—C19—H12	179.8
C4—C5—C6—H2	−178.1	H11—C18—C19—C21	179.8
O2—C7—C8—C9	−39.3 (6)	H11—C18—C19—H12	−0.2
O2—C7—C8—C10	134.1 (4)	C18—C19—C21—C12	177.4 (4)
C1—C7—C8—C9	138.8 (4)	C18—C19—C21—C20	−0.4 (7)
C1—C7—C8—C10	−47.8 (6)	H12—C19—C21—C12	−2.6
C7—C8—C9—N1	175.1 (3)	H12—C19—C21—C20	179.6
C7—C8—C9—H4	−4.9	C15—C20—C21—C12	2.5 (6)
C7—C8—C10—N2	−173.6 (3)	C15—C20—C21—C19	−179.6 (4)
C7—C8—C10—H5	6.4	C16—C20—C21—C12	−177.7 (4)
C9—C8—C10—N2	0.8 (4)	C16—C20—C21—C19	0.3 (6)

### Hydrogen-bond geometry (Å, º)

**Table:**

*D*—H···*A*	*D*—H	H···*A*	*D*···*A*	*D*—H···*A*
O1—H3···O2	0.84	1.84	2.570 (4)	144
C10—H5···O3^i^	0.95	2.29	3.219 (6)	166

Symmetry code: (i) −*x*+2, −*y*+1, −*z*.
